# Dryness of Foot Skin Assessed by the Visual Indicator Test and Risk of Diabetic Foot Ulceration: A Prospective Observational Study

**DOI:** 10.3389/fendo.2020.00625

**Published:** 2020-09-08

**Authors:** Georgios S. Panagoulias, Ioanna Eleftheriadou, Nikolaos Papanas, Christos Manes, Zdravko Kamenov, Dragan Tesic, Stavros Bousboulas, Anastasios Tentolouris, Edward B. Jude, Nikolaos Tentolouris

**Affiliations:** ^1^First Department of Propaedeutic Internal Medicine, Medical School, National and Kapodistrian University of Athens, Laiko General Hospital, Athens, Greece; ^2^Second Department of Internal Medicine, Diabetes Center, Democritus University of Thrace, University Hospital of Alexandroupolis, Alexandroupoli, Greece; ^3^Diabetes Center, Papageorgiou General Hospital, Thessaloniki, Greece; ^4^Diabetes Outpatient Clinic, University Hospital Alexandrovska, Medical University—Sofia, Sofia, Bulgaria; ^5^Clinic for Endocrinology, Diabetes and Diseases of Metabolism, Clinical Center of Vojvodina, Medical Faculty, University Novi Sad, Novi Sad, Serbia; ^6^Diabetes Outpatient Clinic, “St. Panteleimon” General State Hospital, Nikaia, Greece; ^7^Tameside Hospital NHS Foundation Trust and University of Manchester, Ashton-under-Lyne, United Kingdom

**Keywords:** diabetes, foot ulcer, indicator plaster method, neuropad, risk, neuropathy disability score, vibration perception

## Abstract

**Research Question:** Previous cross-sectional studies have shown an association between sudomotor dysfunction and diabetic foot ulceration (DFU). The aim of this prospective multicenter study was to determine the role of dryness of foot skin and of established neurological modalities in the prediction of risk for foot ulceration in a cohort of individuals with diabetes mellitus (DM).

**Design:** The study was conducted from 2012 to 2017. A total of 308 subjects with DM without history of DFU or critical limb ischemia completed the study. Diabetic neuropathy was assessed using the neuropathy symptom score (NSS) and neuropathy disability score (NDS). In a subset of participants, vibration perception threshold (VPT) was evaluated. Dryness of foot skin was assessed by the visual indicator plaster method (IPM). The diagnostic performance of the above neurological modalities for prediction of DFU was tested by receiver operating characteristic curve (ROC) analysis.

**Results:** During the 6-year follow-up, 55 patients (annual ulceration incidence 2.97%) developed DFU. Multivariate Cox-regression analysis after controlling for the effect of age, gender, and DM duration demonstrated that the risk (hazard ratio, 95% confidence intervals) of DFU increased significantly with either abnormal IPM (3.319, 1.460–7.545, *p* = 0.004) or high (≥6) NDS (2.782, 1.546–5.007, *p* = 0.001) or high (≥25 volts) VPT (2.587, 1.277–5.242, *p* = 0.008). ROC analysis showed that all neurological modalities could discriminate participants who developed DFU (*p* < 0.001). IPM testing showed high sensitivity (0.86) and low specificity (0.49), while high vs. low NDS and VPT showed low sensitivity (0.40 and 0.39, respectively) and high specificity (0.87 and 0.89, respectively) for identification of patients at risk for DFU.

**Conclusion:** Dryness of foot skin assessed by the IPM predicts the development of DFU. IPM testing has high sensitivity, whereas high NDS and VPT have high specificity in identifying subjects at risk for DFU. The IPM can be included in the screening methods for identification of the foot at risk.

## Introduction

Diabetic foot ulcers (DFU) are the main cause of amputations, affecting quality of life, and increasing morbidity, mortality and costs (1–3). The global DFU prevalence is on average 5.5% and the annual incidence is ~2% (4–6). Ulcer prevention in people with diabetes mellitus (DM) is of the utmost importance for preventing amputation and reducing the burden on patients and the healthcare system.

Chronic peripheral somatosensory polyneuropathy (DPN) and peripheral arterial disease (PAD) are the main risk factors for development of DFU (4, 6). The American Diabetes Association (ADA) and the International Working Group on the Diabetic Foot (IWGDF) recommends regular examination of people with DM for the diagnosis of DPN and loss of protective sensation using simple standard tests for the identification of those at risk for DFU (4, 7, 8).

Sudomotor dysfunction as a result of damage of the post-ganglionic sympathetic nerves may develop early in the course of DM and result in dry skin and callus formation that can be aggravated by increased plantar pressures caused by foot deformities and DPN (6, 9, 10). Moreover, peripheral autonomic neuropathy results in opening of the arterio-venous shunts, increase of temperature in the feet, and reduced tissue oxygenation (11, 12); such alterations may contribute to the development and delay in healing of DFU. The consensus panel on diabetic neuropathies suggests that sudomotor dysfunction should be evaluated in subjects with DPN and contributes to the detection of autonomic dysfunction (13, 14); however, the lack of simple and widely available equipment has restricted the study of sudomotor function and its contribution to DFU (15). The visual indicator plaster method (IPM) (Neuropad®, TRIGOcare International GmbH, Wiehl, Germany) is a test for the assessment of dryness of foot skin which is a manifestation of sudomotor dysfunction (16, 17). IPM is easy to be performed, is characterized by high sensitivity and negative predictive value for the diagnosis of DPN (16–20) and is proper for self-testing (21).

Previous cross-sectional studies reported a strong association between sudomotor dysfunction or skin dryness and DFU (22, 23). Sudomotor dysfunction assessed by the sympathetic skin response was evident in 90% of the patients with DFU as compared to 33% of the patients without DPN and 47% of those with DPN but without DFU (22). Moreover, dryness of the skin of the feet assessed by the IPM was present in 95% of the patients with DFU as compared to 52.3% of those without DFU (23). However, there are no prospective data on the potential association between testing with the IPM and development of DFU in subjects with DM.

Our research hypothesis herein was that dryness of the skin of the feet acting *per se* through local mechanism and/or reflecting damage to the peripheral sympathetic nervous system is related to the future development of DFU. The primary outcome of this study was to examine the association between dryness of foot skin assessed by the IPM and risk for DFU. Secondary outcomes were to study the diagnostic performance of IPM and other established neurological modalities for the prediction of foot ulceration.

## Materials and Methods

The study was a prospective, multicenter, observational study that was conducted at seven outpatient diabetes clinics in four European countries (Bulgaria, Greece, Serbia, and United Kingdom) in accordance with the Helsinki Declaration of Human Rights (24). The study was approved by the respective ethics committees of the participating hospitals. All patients were examined at baseline visit and were followed-up until the end of the study.

Recruitment started in January 2012 and ended in December 2017. Participants were consecutive adult subjects who attended the outpatient diabetes clinics of the participating hospitals and provided written informed consent. Exclusion criteria were patients having a history of previous ulceration/amputation, Charcot foot, critical limb ischemia, stroke, causes of neuropathy other than DM, severe liver or kidney diseases, current or past history of malignancies other than local basal cell carcinoma, uncontrolled thyroid disease, dermatological diseases that can cause dry skin (psoriasis, scleroderma, contact dermatitis, atopic dermatitis, and seborrheic dermatitis), and recent (within 2 weeks) local application of hydrating lotions, creams, and gels. Critical limb ischemia was defined as a condition characterized by chronic ischemic at rest pain, ulcers, or gangrene in one or both legs attributable to objectively proven arterial occlusive disease (25).

All participants were examined at baseline visit and measurements were performed in constant room temperature of 22–25°C. They were asked to take off their shoes and socks for at least 10 min before examination. Retinopathy status was assessed from the medical records and patients were classified as having or not having retinopathy. Data were also collected for the presence of coronary artery disease (CAD) (stable angina, history of myocardial infarction or of revascularization procedures in the coronary arteries).

The assessment of DPN was based on individuals' history and physical examination by experienced and trained diabetologists. Symptoms were assessed using the Neuropathy Symptom Score (NSS) that examines the presence of pain, cramps or aching in the feet (26, 27). Signs of DPN were assessed using the Neuropathy Disability Score (NDS) that is based on the examination of ankle reflexes, temperature sensation, vibration perception, and pinprick (26, 27). Diagnosis of DPN was based on the following criteria: presence of mild neuropathic signs (NDS = 3–5) with moderate neuropathic symptoms (NSS ≥ 5) or moderate neuropathic signs (NDS ≥ 6) irrespective of neuropathic symptoms (27).

In addition, vibration perception threshold (VPT) at the tip of the great toe of both feet was assessed with a biothesiometer (Bio-Medical Instrument Company, Cleveland, OH, USA) in four out of six outpatient diabetes clinics and data for VPT values were available for 210 participants who completed the study.

Skin dryness was assessed by the IPM. The indicator plaster was applied in the lying position between the first and the second metatarsal head on the plantar surface of both feet and removed after 10 min to evaluate the color change; in the case a callus was present, then it was applied to the nearest non-callused plantar surface. The IPM was evaluated as normal if both plasters changed color from blue to pink and abnormal if at least one plaster remained blue or patchy (16, 17, 21).

Regular follow-up visits to the outpatient clinics were scheduled for all participants every 3–6 months. Subjects were instructed to attend immediately to the outpatient foot clinic of the participating hospital if any foot injury occurred and ulcer evaluation was undertaken. Foot ulcer was defined as any full thickness lesion of the skin at least extending through the subcutis, which could involve muscle, tendon, bone, and joint distal to the malleoli (28). For the purposes of this study, the first ulcer was evaluated in case a patient reported more than one ulcer during follow-up. If a person did not come for examination to the scheduled visits, telephone communication was made either with the patient himself or with a person in his family or with a close relative; they were asked about their health status and whether and when they had ulcers in the feet. If no information could be obtained or if people did not want to answer, they were considered as lost to follow-up and excluded from analysis. Last follow-up visit was the year of ulcer development or 31st December 2017, whichever came first.

### Statistical Analysis

The Statistical Package for the Social Sciences (SPSS software version 22.0 for Windows, Armonk, NY, USA) and the Medcalc Software (version 12.2.1.0, Medcalc, Ostend, Belgium) were used for the analyses. Data were tested for normal distribution of the values using the Kolmogorov–Smirnov test. The values of the normally distributed data are shown as mean ± SD; those without normal distribution are shown as median values (interquartile range), while of categorical data as *n* (%). For comparison of baseline variables between groups, χ^2^ tests were performed for categorical data, Student's *t*-tests were carried out for parametric data and Mann–Whitney *U*-tests were used for non-parametric data.

To assess the relationship between the baseline variables and incidence of foot ulceration, univariate Cox's proportional hazards regression analysis was carried out. Afterwards, multivariate analyses were performed to examine for independent predictors of foot ulceration. Because we found strong interaction between NDS and IPM (*p* = 0.001) as well as VPT (*p* = 0.022), these variables were entered in the models of multivariate Cox-regression analysis separately.

Since previous data demonstrated that an NDS value ≥6 (high NDS) predicts DFU development over a 2-year period (29) and VPT values ≥25 Volts (high VPT) also predicts DFU in cross-sectional (30) and prospective data (31), we used NDS and VPT as both continuous and categorical variable in the analyses.

The time to foot ulceration was evaluated by Kaplan–Meier estimates; equality of the survivor functions between the groups stratified according to the studied neurological modalities was estimated using the log-rank test.

We calculated and compared the areas under the receiver operating characteristic (ROC) curves of the IPM result (abnormal vs. normal), high NDS, and high VPT regarding their ability to discriminate those who developed vs. those who did not develop foot ulcers. In addition, we calculated the diagnostic performance [sensitivity, specificity, positive (+LR), and negative likelihood ratios (−LR) and positive (+PV) as well as negative (−PV) predictive values] of these variables for the prediction of foot ulceration. *P* < 0.05 (two-sided) were considered statistically significant.

## Results

### Screened Cohort

A total of 381 consecutive subjects were screened; 14 patients did not meet the inclusion criteria and were excluded; from the 367 subjects enrolled, 18 (4.9%) patients died before DFU development and 41 (11.2%) were lost to follow-up (Supplementary Figure 1). Subjects deceased and lost to follow-up did not differ from those who completed the study in terms of age (62.12 ± 10.57 vs. 62.81 ± 11.28 years, respectively, *p* = 0.672), gender (male/female 32/27 vs. 153/155, respectively, *p* = 0.521), diabetes duration [10.0 (4.0–18.0) vs. 11.50 (4.0, 20.0) years, respectively, *p* = 0.563], presence/absence of DPN (35/24 vs. 159/149, respectively, *p* = 0.278), retinopathy (0/39 vs. 102/206, respectively, *p* = 0.907) or CAD (17/42 vs. 90/218, respectively, *p* = 0.950).

The baseline clinical characteristics of the study participants who completed the study and were used in the analyses are shown in Table 1. Patients who developed DFU were older (*p* = 0.030), had longer diabetes duration (*p* = 0.045), more often DPN (*p* < 0.001) and worse all neurologic modalities (*p* < 0.05) and had more often retinopathy (*p* < 0.001) as well as CAD (*p* = 0.009).

**Table 1:** Demographic and clinical characteristics of the study subjects.

	**Developed DFU (*n* = 55, 17.9%)**	**Not developed DFU (*n* = 253, 82.1%)**	***p***
Male/female *n* (%)	32 (58.2)/23 (41.8)	121 (47.8)/132 (52.2)	0.164
Age (years)	65.7 ± 11.2	62.0 ± 1.3	0.03
Type 1/type 2 diabetes *n* (%)	3 (5.5)/52 (94.5)	17 (6.7)/236 (93.3)	0.73
Diabetes duration (years)*	15 (4, 23)	10 (4, 17)	0.045
Any retinopathy *n* (%)	31 (56.4)	71 (28.1)	0.009
CAD *n* (%)	24 (43.6)	66 (26.1)	<0.001
DPN status *n* (%)			
DPN−	13 (23.6)	163 (64.4)	
DPN+	42 (76.4)	90 (35.6)	<0.001
NSS*	6.5 (3, 8)	5 (0, 7)	0.017
NDS*	6 (4, 6)	2 (0, 4)	<0.001
High NDS *n* (%)	22 (40.0)	34 (13.4)	
Low NDS *n* (%)	33 (60.0)	219 (86.6)	<0.001
Results with IPM testing *n* (%)			
Normal IPM	7 (12.7)	121 (47.8)	
Abnormal IPM	48 (87.3)	132 (52.2)	<0.001
VPT (Volts)*	6 (0, 33)	10 (6, 18)	0.776
High VPT	15 (34.9)	22 (13.2)	
Low VPT	28 (65.1)	145 (86.8)	0.001

Data are shown as mean ± SD or n (%).

* Median value (interquartile range).

CAD, coronary artery disease; NDS, neuropathy disability score; NSS, neuropathy symptom score; IPM, indicator plaster method; high NDS, neuropathy disability score ≥6, low NDS, neuropathy disability score <6; high VPT, vibration perception threshold ≥25 Volts; low VPT, vibration perception threshold <25 Volts.

*Data for VPT are from 210 participants*.

### Factors Predicting Development of Foot Ulcers

In total, 55 out of the 308 patients developed DFU during the 6-year follow-up period, giving an overall ulceration incidence of 17.85% or an average annual ulceration incidence of 2.97%.

Univariate Cox-regression analysis showed that the risk for foot ulceration increased significantly with presence of DPN (*p* = 0.030), IPM result (abnormal vs. normal) (*p* < 0.001), NDS used as a continuous variable (*p* < 0.001), NDS used as a categorical variable (high NDS vs. low NDS, *p* < 0.001 and NDS = 3–5 vs. NDS < 3, *p* = 0.006), VPT used as a continuous variable (*p* = 0.040), and VPT used as categorical variable (high VPT vs. low VPT) (*p* = 0.001). There was a trend for association between diabetes duration and risk of foot ulceration (*p* = 0.057); no significant associations were found with age, gender or NSS (Table 2).

**Table 2:** Predictive variables for development of foot ulcers byunivariate Cox's proportional hazards regression models.

**Variable**	**Number of participants**	**HR**	**95% CI**	***p***
**Gender**				
Female	155	1.00	–	
Male	153	1.39	0.82–2.84	0.224
Age (years)	308	1.02	0.99–1.05	0.104
Diabetes duration (years)	308	1.08	0.99–1.04	0.057
DPN status	308			
No DPN	176	1.00	–	
With DPN	132	1.11	1.01–1.22	0.03
NSS as continuous variable	308	1.05	0.96–1.57	0.226
NDS as continuous variable	308	1.35	1.22–1.49	<0.001
**NDS as categorical variable** **(****1****)**				
Low NDS	252	1.00	–	
High NDS	56	3.1	1.81–5.32	<0.001
**NDS as categorical variable** **(****2****)**				
NDS = 0–2	111	1.00		
NDS = 3–5	143	3.799	1.45–9.84	0.006
**Results with IPM testing**				
Normal result	128	1.00	–	
Abnormal result	180	4.57	2.07–10.11	<0.001
VPT as continuous variable (Volts)	210	1.02	1.00–1.04	0.04
**VPT as categorical variable**				
Low VPT	173	1.00	–	
High VPT	37	3.02	1.61–5.67	0.001

DPN, diabetic peripheral neuropathy; NDS, neuropathy disability score; NSS, neuropathy symptom score; IPM: indicator plaster method; high NDS, neuropathy disability score ≥6; low NDS, neuropathy disability score <6; VPT, vibration perception threshold; high VPT, vibration perception threshold ≥25 Volts; low VPT, vibration perception threshold <25 Volts.

*Data for VPT are from 210 participants*.

Multivariate Cox-regression analysis after controlling for the effect of age, gender, and diabetes duration demonstrated that the risk [hazard ratio, 95% confidence intervals (CI)] of foot ulceration increased significantly with either abnormal IPM result (3.319, 1.460–7.545, *p* = 0.004) or high NDS (2.782, 1.546–5.007, *p* = 0.001) or high VPT (2.587, 1.277–5.242, *p* = 0.008). No significant independent relationship was found between mild vs. no neuropathic signs (NDS = 3–5 vs. NDS <3) or with presence/absence of DPN and risk of foot ulceration.

**Figure 1:**
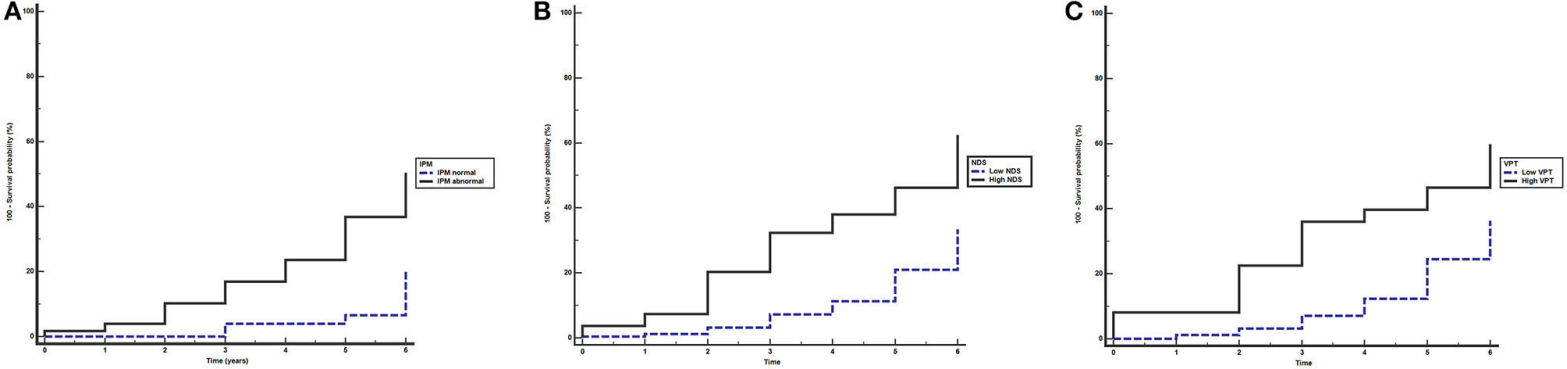
Kaplan–Meier estimates of foot ulceration in participants during the study period. Equality of the survivor functions between the groups stratified by different neurological modalities was performed using the log-rank test. **(A)** abnormal (continuous line) vs. normal (dashed line) indicator plaster method (IPM) **(A)**: *X*^2^ = 18.364, *p* < 0.001; **(B)** high (≥6) (continuous line) vs. low (<6) (dashed line) neuropathy disability score (NDS): *X*^2^ = 20.291, *p* < 0.001; **(C)** high (≥25) (continuous line) vs. low (<25) (dashed line) vibration perception threshold (VPT) (Volts): *X*^2^ = 14.079, *p* < 0.001.

The median (95% CI) follow-up time was 3.0 (3.0–4.0) years. Kaplan–Meier analysis showed that the proportion of participants who developed DFU during the study was significantly higher for those having abnormal vs. normal IPM result, high vs. low NDS, and high vs. low VPT (all *p* < 0.001; Figure 1).

ROC analysis showed that the areas under the curves of IPM result, NDS, and VPT status could discriminate significantly (*p* < 0.05) the participants who developed DFU (Table 3).

**Table 3:** The performance (value, 95% confidence intervals) of the tests used in the study for the diagnosis of patients who developed foot ulcers.

**Variable curve**	**Area under the ROC**	***p***	**Sensitivity**	**Specificity**	**+LR**	**−LR**	**Youden index J**
IPM	0.675 (0.620–0.727)	<0.001	0.87 (0.87–0.95)	0.49 (0.42–0.54)	1.67 (1.4–2.0)	0.27 (0.1–0.5)	0.351
High NDS	0.635 (0.578–0.698)	0.001	0.40 (0.27–0.54)	0.89 (0.82–0.91)	3.05 (1.9–4.8)	0.69 (0.6–0.9)	0.269
IPM and high NDS	0.637 (0.580–0.691)	0.023	0.40 (0.27–0.54)	0.87 (0.83–0.91)	3.16 (2.0–5.0)	0.69 (0.6–0.9)	0.273
IPM or high NDS	0.664 (0.609–0.717)	<0.001	0.85 (0.73–0.96)	0.47 (0.41–0.54)	1.63 (1.4–1.9)	0.31 (0.2–0.6)	0.328
High VPT	0.689 (0.539–0.657)	0.035	0.39 (0.21–0.51)	0.87 (0.81–0.92)	2.65 (1.5–4.7)	0.75 (0.6–0.9)	0.217
IPM and high VPT	0.606 (0.536–0.672)	0.04	0.33 (0.19–0.49)	0.89 (0.83–0.93)	2.86 (1.6–5.2)	0.76 (0.6–0.9)	0.211
IPM or high VPT	0.660 (0.592–0.724)	0.0001	0.91 (0.78–0.97)	0.41 (0.34–0.49)	1.55 (1.3–1.8)	0.23 (0.1–0.6)	0.32
IPM and NDS = 3–5	0.713 (0.659–0.763)	<0.001	0.84 (0.71–0.92)	0.59 (0.53–0.65)	2.03 (1.7–2.5)	0.28 (0.2–0.5)	0.425

*ROC, receiver operating characteristic; IPM, indicator plaster method; +LR, positive likelihood ratio; −LR, negative likelihood ratio; NDS, neuropathy disability score; high NDS, neuropathy disability score ≥6; VPT, vibration perception threshold; high VPT, vibration perception threshold ≥25 Volts; NDS and IPM, combined variable of participants with both tests abnormal; high NDS or IPM, combined variable of participants with abnormal either high NDS or IPM test; high VPT and IPM, combined variable of participants with both tests abnormal; high VPT or IPM, combined variable of participants with abnormal either high VPT or IPM test; NDS = 3–5 and IPM: combined variable of participants with both mild neuropathic signs and abnormal IPM*.

The IPM showed high sensitivity (0.86) and a low specificity (0.49) for identification of patients at risk. On the other hand, high NDS and high VPT showed low sensitivity (0.40 and 0.39, respectively) but high specificity (0.87 and 0.89, respectively) for identification of patients at risk of DFU. The effect on posttest probability for the development of DFU (+LR) was slight for IPM (1.67), and slight to moderate for high NDS (3.05) as well as for high VPT (2.65) (32). The effect on posttest probability for absence of DFU (−LR) was moderate for IPM (0.27), and slight for high NDS (0.69) as well as for high VPT (0.75) (Table 3) (32). The IPM alone or in combination with either high NDS or high VPT had high −PV (>0.90) but low +PV (<0.30) for identification of patients at risk of DFU (Supplementary Table 1).

We created new variables from the combination of IPM and/or high NDS, IPM and/or high VPT, high NDS and/or high VPT, as well as IPM and mild neuropathic signs (NDS = 3–5) aiming to examine the performance of the new combined variables for the diagnosis of patients who developed DFU; however, the new variables did not change much their performance compared to the individual variables that constituted them (Table 3).

In addition, pairwise comparisons of the areas under the ROC curves of the IPM, high NDS, high VPT, mild neuropathic signs, and their combinations did not differ significantly (all *p* > 0.05; Supplementary Table 2).

### Location of the Ulcers, Amputations, and Deaths

A total of 32 of the ulcers occured on the right foot (18 toe ulcers, 3 heel ulcers, and 11 ulcers under the metatarsal heads) and 23 on the left foot (13 toe ulcers, 2 heel ulcers, and 8 ulcers under metatarsal heads). During the follow-up period, there were seven cases with amputations (six minor and one below the knee amputation) giving an overall amputation incidence of 2.27% for the 6-year period or an average annual amputation incidence of 0.37%. Regarding the cause of death of the deceased patients, they were mainly deaths due to cardiovascular diseases (*n* = 10), malignancy (*n* = 4), and sepsis (*n* = 4) not related to diabetic foot infections.

## Discussion

This study has shown that dryness of foot skin assessed by the IPM is an independent predictor of risk of foot ulceration in individuals with DM. In addition, it was confirmed that other neurological modalities such as high NDS and high VPT are also associated with increased risk of foot ulceration.

The burden of diabetic foot disease is enormous and the need for effective strategies to prevent foot ulceration imperative. The lifetime incidence of foot ulcers in people with diabetes has been previously suggested to be 15–25% (33) or even higher (19–34%) according to recent estimates (6). DFUs have been shown to precede amputation in up to 85% of cases in people with diabetes (34). The risk of death at any time point for patients with DFU is 3 times higher in comparison with people with diabetes who do not have DFU (35). Moreover, 30–40% and up to 65% of the healed DFU recur in the 1st year and within 6 years, respectively, after an ulcer episode (6). Therefore, identifying the at-risk patient is probably the most important step in reducing the rate of first DFU development and its devastating consequences.

According to current guidelines, various validated methods have been proposed and used in daily practice to detect people at risk for DFU including 5.07 Semmes–Weinstein monofilament (SWF) testing, the Ipswich touch test, clinical examination of pinprick, temperature and vibration sensation together with ankle reflexes (NDS), evaluation of VPT, and assessment of peripheral arterial blood flow (7, 8). However, no single gold-standard examination exists for the prediction of DFU and all neurological modalities proposed and used for the diagnosis of patients at risk have their advantages and their limitations that should be understood by the requesting health care provider. It should also be emphasized that each neurological modality assesses different aspects of diabetic neuropathy, since other tests assess small fiber integrity and other tests large fiber integrity. Sudomotor dysfunction as a result of small fibers damage develops early in the course of DM before large fiber damage and sensory loss (9, 10, 36, 37). SWF assesses large fiber function, the Ipswich touch test both small and large fiber function, pinprick and temperature tests assess small fiber function and vibration tests assess large fiber function. NDS has the advantage of including tests assessing both small and large fiber, whereas VPT assesses large fiber function. On the other hand, quantitative sudomotor axon reflex test (QSART) is considered the reference method for the detection of sudomotor dysfunction, but it is time-consuming and cannot be easily used in every day clinical practice, while its availability is still limited. Thus, NDS and VPT were used in our study and their performance was compared with IMP for the prediction of foot ulceration.

Neurological examination has been evaluated in a large prospective study of 2 years duration involving 9,710 with diabetes in the primary care setting in the UK (29). The authors found an annual incidence of new DFU of 2.2% in the general diabetes population including patients with present or past DFU, which is comparable with the incidence found in our study (2.97%). In addition, they showed that participants with NDS ≥ 6 had a relative risk (RR) of 2.32 for DFU, independently of other factors like gender, diabetes duration, NSS, footwear, and comorbidities such as nephropathy or retinopathy. Notably, we found that an NDS ≥ 6 was independently associated with increased risk for DFU in our cohort with a HR 2.78. Another prospective multicenter study reported that in a cohort of 248 patients with diabetes attending diabetic foot clinics for various reasons, high NDS (NDS ≥ 5) was associated independently with increased risk of DFU over a period of 30 months in multivariate analysis with an OR of 3.1 (1.3–7.6) (38). Regarding the performance of NDS ≥ 5 for the prediction of DFU, the authors reported that high NDS had sensitivity 0.92, specificity 0.43, and +PV 0.28. We found that an NDS ≥ 6 had low sensitivity (0.40) and +PV (0.40) but high specificity (0.89) and −PV (0.87) for DFU prediction. The differences could be attributed to the different populations included in the two studies. Pham et al. included patients at higher risk for DFU when compared with our participants, a notion that is further reinforced by the high annual incidence of DFU (11.6%) observed in that study (38). Moreover, our patients were older and could have had absent ankle reflexes and thus higher NDS values due to age or reasons other than DPN.

Using the same methodology, Pham et al. (38) also found an independent association between high VPT (≥25 V) and development of DFU, which is in agreement with the results of this study. With regards to high VPT, the authors reported that it had a sensitivity of 0.86, a specificity of 0.56, and a +PV of 0.32 in the prediction of DFU (38). In the present study, we found that high VPT had a sensitivity of 0.39, a specificity of 0.87, a +PV of 0.41, and a –PV of 0.84. Young et al. (31) found that high VPT (>25 V) vs. VPT <15 was associated with a 6.8-fold increased risk of developing DFU over a 4-year period. A systematic review of the studies by Pham and Young showed that high VPT (>25 V) had a sensitivity of 0.83–0.86, a specificity of 0.57–0.63, a +PV of 0.20–0.32, a −PV of 0.95–0.97, a +LR of 2.0–2.2, and a –LR of 0.3 for the prediction of DFU (33). Nevertheless, it should be emphasized that VPT is affected by age and many patients with VPT values <25 V may have small fiber neuropathy and thus be at risk of DFU (39).

The difference in the performance of high NDS and high VPT in prediction of DFU between this study and the previous studies is most likely related to differences in the prevalence of established neuropathy in the studied populations; Pham et al. (38) included 35.1% of patients with previous DFU, while 60% of the participants had more severe neuropathy with high NDS and 52.4% had high VPT, while 43.1% in the study by Young et al. (31) had also high VPT. In this study, we excluded patients with history of DFU and a small number of participants had high NDS (18.2%) and high VPT (12.0%).

The use of SWM has been shown to predict future development of DFU (33, 40) and according to current guidelines is recommended for identification of people with loss of protective sensation and at risk for DFU (7, 8). Nevertheless, it should be emphasized that SWM detects advanced large fiber neuropathy and the current recommendation for the assessment of DPN using SWM testing may need to be reconsidered (36, 37). A systematic review and meta-analysis demonstrated that in 3 prospective studies, SWM had a sensitivity 0.66–0.91, a specificity 0.34–0.86, a +PV 0.18–0.39, a −PV of 0.94–0.94, a +LR of 1.4–4.7, and a −LR of 0.3–0.5 (33). However, it should be noted that clear recommendations regarding on which sites of the foot it should be tested and how many false results are considered abnormal became only recently available (7). Hence, the interpretation of the results has been confusing for the health care professionals and the diagnostic performance of the test undermined. This was the reason why SWM has not been evaluated in this study. Although the first guidelines by the IWGDF were published in 1999 (27), not many European health care professionals were familiar with the technique and many of them used to perform the test in 3–10 plantar positions and to grade and evaluate the result as normal/abnormal differently. Thus, we decided not to include SWM testing at the beginning of this study.

Another test recommended for the detection of loss of protective sensation is the Ipswich touch test (7). It is a simple, cheap, and reproducible amongst both healthcare professionals and patients, making it an excellent tool for the diagnosis of established neuropathy (41, 42). It has been evaluated in cross-sectional, but not in prospective studies, and has shown a sensitivity of 0.76–0.79, a specificity of 0.90, a +PV of 0.89–0.90, a −PV of 0.77–0.79, a +LR of 7.7–8.1, and a −LR of 0.24–0.27 for the identification of patients with established neuropathy, as defined by VPT ≥ 25 V (42); however, no studies exist regarding the association between abnormal test results and development of DFU. Nevertheless, the Ipswich touch test remains a useful tool for the detection of patients with advanced neuropathy. The Ipswich touch test has not been evaluated in this study because it was developed and evaluated early in the 2010s and it was not included in the guidelines for screening purposes when this study was performed.

Regarding nerve conduction studies, one study examined the role of common peroneal motor nerve conduction velocity (MNCV) in the prediction of DFU (43). A total of 169 people with diabetes were followed up over a 6-year period and it was reported that a reduced MNCV was an independent predictor of increased risk for DFU. Nevertheless, nerve conduction studies are not widely available, need to be performed by experienced personnel and cannot be recommended as a screening tool for detecting DPN and foot at risk.

Similar reasons preclude the use of QSART, the thermoregulatory sweat test, and the quantitative direct and indirect reflex test (QDIRT) that assess sudomotor dysfunction for the prediction of DFU (14). Sudoscan, on the other hand, is a quick and easy method for assessing sweat function and thus sudomotor function, although the device is not widely available (37). Nevertheless, to our knowledge, no data is available so far for the performance of these diagnostics techniques for the prediction of DFU.

In the present study, we found that an abnormal IPM result was an independent predictor of DFU. In addition, we found that despite differences in sensitivity and specificity, the overall performance of IPM was comparable with other established tests like high NDS and high VPT for the prediction of DFU. The IPM had high sensitivity and −PV but low specificity and +PV. The effect on posttest probability for the development (+PV) of DFU was slight for IPM (1.67), while the effect on posttest probability for the absence (−PV) of DFU was moderate (0.27) (32). From the clinical point of view, a high sensitivity is clearly important whenever a test like IPM is aimed to be used in the general diabetes population to identify patients at risk for DFU. However, the test has lower specificity which means that a high proportion of people with diabetes and a positive IPM testing will not develop DFU. Nevertheless, what is required in the diabetes community is to have simple, fast, inexpensive, widely available, and reliable tests for screening in the primary care setting for people at risk of DFU. Undoubtedly, an ideal test must have both high sensitivity and specificity (44). However, most tests used in daily clinical practice have either high sensitivity and lower specificity or vice versa (44). A test aiming to recognize people with diabetes at risk for developing DFU should have high sensitivity and, regardless of its specificity, should not expose the patient who has a positive result to potential risks by performing further invasive or other tests that have high specificity but they may be harmful (44). In the case of a positive IPM, patients with diabetes will be further examined clinically for confirmation of loss of protective sensation and if the diagnosis is verified they will have their foot examined at least twice a year according to their risk for foot ulceration, they will be educated about foot care and appropriate footwear, and will receive ongoing professional foot care when needed (7). However, a negative IPM test means that we can conclude with 94% certainty that the patient is at low risk of developing a DFU in the frame of the population with the characteristics examined in and the duration of this study.

Furthermore, in the present study we investigated whether combining two different screening tests, for example, IPM—a test with high sensitivity—together with either high NDS or VPT—tests with high specificity—would alter the performance of the new combined modality for the prediction of DFU development. However, this approach did not alter much nor significantly the performance of the combined modalities compared to the individual tests. It should be noted that none of the tests used in our study or any combination of tests had high performance for the prediction of DFU (Table 3). This is due to the multifaceted etiology of DFU and although DPN is beyond doubt one of the major risk factors for the development of DFU, PAD, foot deformities, poor glycemic control, and other microvascular complications also play significant roles.

Screening for patients at risk for DFU can be identified using several different tools according to the IWGDF/ADA guidelines (7, 8). However, the adoption and implementation of the recommendations in clinical practice is rather disappointing and a National Health Survey in 2010 in the United Kingdom reported that more than 50% of individuals with diabetes do not recollect having their feet examined or being given any advice regarding foot care (42). One recent study examined perception and knowledge of the pathogenesis and management of DFU among 600 general practitioners (GPs) in four countries in Europe–France, United Kingdom, Spain, and Germany (150 GPs per country) (45). A total of 1,188 patient cases from the four countries were also collected. The authors reported that more than 80% of GPs mentioned neuropathy “often” or “quite often” as an important causative factor in the development of DFU. However, only 30% of the GPs in France, 39% in the United Kingdom, 41% in Spain, and 80% of the GPs in Germany performed any neurologic testing in people with diabetes. The respective proportion per county of GPs who performed clinical examination for neuropathy was 11, 26, 15, and 38%, respectively; SWM testing 16, 9, 20, and 15%, respectively; and vibration testing 1, 3, 5, and 43%, respectively (45). An older study from the USA assessed adherence to diabetes guidelines in the seven primary care physicians practices in the county (46). The performance of primary care physicians was assessed at baseline and after 1 year of local consensus guidelines development and implementation of various interventions. Before any intervention, rate of adherence to guidelines for foot examination was only 15% and increased to 42% one year after intervention. The findings of these studies underline the big gap between guidelines and daily practice even in countries with long-standing integrated foot care services. A one-stop service for screening all diabetic microvascular complication and both small and large fiber neuropathy may increase early DPN diagnosis and appropriate foot screening and lead to improved clinical outcomes (47).

The IPM has been evaluated extensively for the diagnosis of neuropathy and has been found to have good sensitivity (65–100%) and modest specificity (32–79%) (16–20, 37). IPM testing is easy to be performed by all healthcare professionals and even by the patients themselves (21). IPM result can be characterized as normal, patchy or abnormal or can be expressed as a continuous variable estimated as the percentage color change of the IPM in pink (48). Although the latter way of assessing IPM may increase its diagnostic performance, it remains subjective until an image analysis software is developed. One drawback of the IPM test is its cost. However, a recent study showed that using the IPM together with SWM as triage tests for DPN and its late sequelae is an optimal strategy when compared with the approach of using SWM alone, leading to significant cost-savings and health gains (49). In this study it was demonstrated that the IPM testing predicts DFU and has a performance similar to that of established tests widely used and recommended for identification of people at risk; in addition, it has high sensitivity and −PV. Although most available tests for assessing DPN are simple, quick, and inexpensive, the growing number of patients with diabetes and the limited time of primary care physicians turn broad diabetic foot screening in the community into a complex and time-consuming procedure. The benefit of IPM is that it is proper for self-testing and can be performed by the patient at home provided that he has been given clear written instructions on how to perform and evaluate the result. Although in our study health care professionals applied the IPM and our results may not have been reproduced if self-testing was performed, the overall agreement between patient and health care provider application of the IPM has been reported over 90% (21). Moreover, it should be noted that around 20% of older people with visual and/or kinetic impairment requested help for self-testing (21). However, given the poor compliance of physicians with feet examination for identification of those at risk of ulceration, self-testing approach could significantly increase the number of people who would have been screened and at the same time would save time for health care professionals. It should be emphasized that identification of people at risk is the first step in preventing DFU. After diagnosis, several interventions if applied and adopted by people with diabetes could reduce the risk of developing the first ulcer (7, 50). Another advantage of the IPM self-testing is that patients may feel more engaged and in control of their condition thus improving their compliance with the appropriate foot care, while the visual confirmation of IPM may make them more aware of their risk of ulceration.

Regarding the role of the underlying mechanisms involved in the development of DFU and the contribution of the IPM in the detection of them, only assumptions can be made by this study. Foot sweat glands are innervated by sudomotor unmyelinated cholinergic nerve fibers and sudomotor dysfunction is considered a manifestation of peripheral autonomic neuropathy (15). However, it should be emphasized that diabetic neuropathy involves both somatic and sympathetic components of peripheral nerve fibers and both small and large nerve fibers (10, 51). Clinical examination and nerve conduction studies evaluate different nerve fibers and mainly assess the somatic function of large myelinated fibers. Previous studies demonstrated that IPM correlated strongly with small fiber neuropathy assessed by quantitative sensory testing, heart rate variability, neuropathic symptoms, and intra-epidermal fiber density in subjects with diabetes and has been suggested as an effective screening instrument for small fiber neuropathy (19), while it was found that it has high sensitivity and lower specificity in comparison with neurological modalities that examine large nerve fibers in the diagnosis of DPN (17). Interestingly, small unmyelinated fibers are the earliest nerve fibers to undergo damage in diabetes and peripheral sympathetic neuropathy may be detectable before conventional autonomic and somatic nerve function tests become abnormal (9, 52). It has been even suggested that small sympathetic fiber dysfunction could be associated more with the development of DFU than large fiber neuropathy (51). Prospective data showed that skin temperature elevation, regulated by microvasculature occurred in parallel with development of foot sweating problems in patients at risk of DFU (51). A recent prospective study assessed the performance of foot temperature monitoring for the prevention and early detection of diabetic foot complications; it was found that over 34 weeks increased (by 2.2°C, 4°F) average foot temperature for at least 2 consecutive days predicted early the development of DFU (53). The authors reported that the performance of this approach for DFU prediction across four ipsilateral temperature range settings was as follows: sensitivity 0.53–0.97, specificity 0.33–0.78, +PV 0.15–0.22, and −PN 0.93–0.99. In a previous study, we found that abnormal SSR response was detected in the vast majority (almost 90%) of the patients with DFU (22). Because sweating and thermoregulatory functions are controlled by the small nerve fibers of the autonomic nervous system, it is likely that the IPM detects early small fiber neuropathy, thus predicting DFU risk. On the other hand, it cannot be ignored that damage of the large myelinated nerve fibers and increased plantar pressures are also important since 58.5% of the DFU in this study were developed in the toes due probably to loss of protective sensation and/or other factors, while 43.5% in the plantar area of the foot due probably to the combination of loss of protective sensation, altered biomechanics, and small fiber neuropathy.

Strengths of this study are as follows: this was a multicenter study in four European countries with different organization of footcare services; in addition, consecutive people attending outpatient clinics of the participated hospitals free of DFU or amputations and not selected or high-risk patients for DFU were recruited; therefore, they can be considered as representative of the general diabetes population; moreover, a relatively large number of participants were recruited and the major outcome was well-defined and evaluated; furthermore, the fact that we found a similar DFU incidence rate to that of other studies gives validity to the results of this study.

Limitations of the study are: selection biases are inevitable due to patient non-attendance for regular review and screening appointments; moreover, we did not include SWM testing which is included in the guidelines for the diagnosis of loss of protective sensation; comparison of the performance of the IPM with SWM testing would be useful and informative; furthermore, the number of events in this study was relatively small, and therefore the findings should be interpreted with caution; and finally the specificity of the test is low but the −PV is high, meaning that many patients with abnormal results may be classified falsely as being at risk, while a normal result excludes safely those at risk; clearly, if IPM is adopted as a single screening tool for DFU prevention, cost-effectiveness data in the real-world setting are required to examine the value of IPM *per se* for the healthcare system.

In conclusion, this is the first prospective study showing that dryness of foot skin assessed by IPM testing predicts the development of DFU. Previous studies demonstrated that the IPM is a simple, reliable, easy to be performed by all health care professionals and sensitive test for identification of the foot at risk; in addition, it is proper for self-testing. If foot at risk is identified in a timely manner and appropriately managed, many DFU could be prevented. Supplementation of IPM in daily clinical practice performed by the patients can save time for physicians and may save limbs in individuals with diabetes.

## Data Availability Statement

The raw data supporting the conclusions of this article will be made available by the authors, without undue reservation.

## Ethics Statement

The study was approved by the respective ethics committees of the participating hospitals [Laiko General Hospital, Athens, Greece; University Hospital of Alexandroupolis, Alexandroupolis, Greece; Papageorgiou General Hospital, Thessaloniki, Greece; University Hospital Alexandrovska, Sofia, Bulgaria; Novi Sad University Hospital, Novi Sad, Serbia; St. Panteleimon General Hospital, Nikaia, Greece; National Research Ethics Service (NRES), NRES Committee North West, Great Manchester North, Manchester, UK] All patients were examined at baseline visit and were followed-up until the end of the study. The patients/participants provided their written informed consent to participate in this study.

## Author's Note

Preliminary data of this work were presented at the 50th Annual Meeting of the European Association for the Study of Diabetes, 2014, Vienna, Austria.

## Author Contributions

GP: collection and interpretation of data and preparation of manuscript. IE and AT: interpretation of data and preparation of manuscript. NP: concept and design, collection of data, and preparation of manuscript. CM, ZK, DT, SB, and EJ: collection of data and preparation of manuscript. NT: concept and design, interpretation of data, and preparation of manuscript. All authors contributed to critically revise the manuscript and have given final approval of the version to be published.

## Conflict of Interest

The authors declare that the research was conducted in the absence of any commercial or financial relationships that could be construed as a potential conflict of interest.
