# Supplementary figures and images for: Dryness of Foot Skin Assessed by the Visual Indicator Test and Risk of Diabetic Foot Ulceration: A Prospective Observational Study
Source: Front Endocrinol (Lausanne). 2020 Sep 8;11:625. doi: 10.3389/fendo.2020.00625 (PMC7506164; doi:10.3389/fendo.2020.00625)

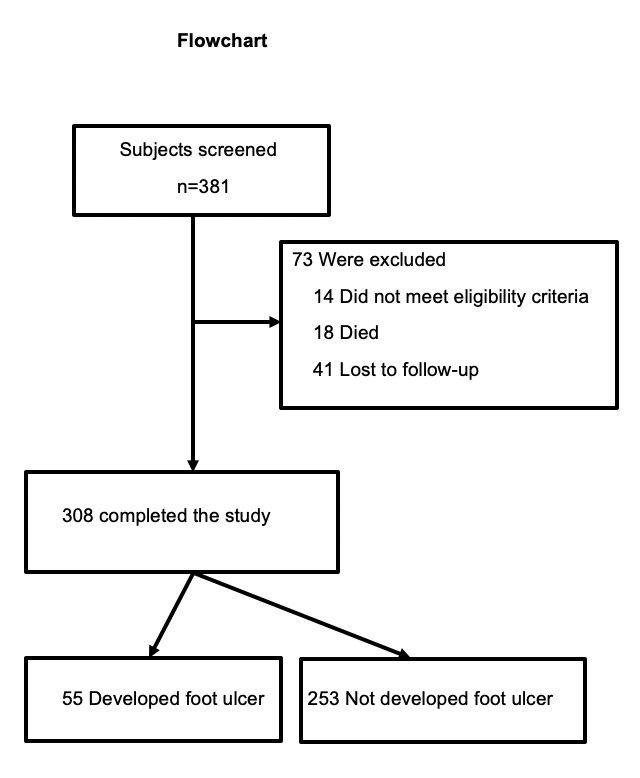

Supplement: Supplementary file 1 [file Image_1.tiff]
