# Supplementary material for: Dryness of Foot Skin Assessed by the Visual Indicator Test and Risk of Diabetic Foot Ulceration: A Prospective Observational Study
Source: Front Endocrinol (Lausanne). 2020 Sep 8;11:625. doi: 10.3389/fendo.2020.00625 (PMC7506164; doi:10.3389/fendo.2020.00625)
Supplement: Supplementary file 2 [file Table_1.docx]

**Supplementary Table 1.**

Positive (+) and negative (-) predictive values of the tests used in the study for the diagnosis of patients who developed foot ulcers

**Variable + Predictive value -Predictive value**

IPM 0.27 (0.24-0.30) 0.94 (0.89-0.97)

High NDS 0.40 (0.30-0.51) 0.87 (0.84-0.89)

IPM and high NDS 0.41 (0.30-0.52) 0.87 (0.84-0.89)

IPM or high NDS 0.26 (0.23-0.29) 0.94 (0.89-0.97)

High VPT 0.41 (0.28-0.55) 0.84 (0.81-0.87)

IPM and high VPT 0.42 (0.29-0.57) 0.84 (0.81-0.86)

IPM or high VPT 0.29 (0.25-0.32) 0.95 (0.87-0.98)

NDS=3-5 and IPM 0.31 (0.27-0.35) 0.94 (0.90-0.97)

IPM: indicator plaster method, VPT: vibration perception threshold, NDS: neuropathy disability score, high NDS: ≥6, high VPT: vibration perception threshold ≥25 Volts.

NDS and IPM: combined variable of participants with both tests abnormal; high NDS or IPM: combined variable of participants with abnormal either high NDS or IPM test; high VPT and IPM: combined variable of participants with both tests abnormal; high VPT or IPM: combined variable of participants with abnormal either high VPT or IPM test; NDS=3-5 and IPM: combined variable of participants with both mild neuropathic signs and abnormal IPM.
