# Supplementary material for: Dryness of Foot Skin Assessed by the Visual Indicator Test and Risk of Diabetic Foot Ulceration: A Prospective Observational Study
Source: Front Endocrinol (Lausanne). 2020 Sep 8;11:625. doi: 10.3389/fendo.2020.00625 (PMC7506164; doi:10.3389/fendo.2020.00625)
Supplement: Supplementary file 3 [file Table_2.docx]

**Supplementary Table 2.**

Comparison of the areas under the receiver and operating characteristic curves (ROC) of the tests used in the study regarding discrimination of patients who developed or not foot ulcers

**Variable** **Difference (mean, 95% CI) p**

**between areas under ROC**

IPM vs.high NDS 0.040 (-0.056, 0.138) 0.419

IPM vs.IPM and high NDS 0.038 (-0.057, 0.135) 0.282

IPM vs.IPM or high NDS 0.011 (-0.013, 0.035) 0.369

High NDS vs.IPM and high NDS 0.001 (-0.019, 0.023) 0.857

High NDS vs.IPM or high NDS 0.029 (-0.065, 0.125) 0.542

IPM andhigh NDS vs. IPM or high NDS 0.027 (-0.068, 0.123) 0.571

IPM vs.high VPT 0.034 (-0.091, 0.160) 0.594

IPM vs.IPM and high VPT 0.036 (-0.083, 0.157) 0.546

IPM vs.IPM or high VPT 0.017 (-0.035, 0.041) 0.892

High VPT vs.IPM and high VPT 0.026 (-0.062, 0.166) 0.375

High VPT vs.IPM or high VPT 0.051 (-0.062, 0.166) 0.375

High VPT or IPM vs. high VPT and IPM 0.054 (-0.061, 0.170) 0.358

High NDS vs. high VPT 0.040 (-0.042, 0.124) 0.339

High NDS vs. IPM and high VPT 0.037 (-0.038, 0.144) 0.332

High NDS vs. high VPT or IPM 0.092 (-0.024, 0.209) 0.122

IPM and NDS=3-5 vs. high NDS 0.105 (-0.007, 0.218) 0.065

IPM and NDS=3-5 vs. high VPT 0.064 (-0.058, 0.188) 0.310

IPM and NDS=3-5 vs. IPM and high NDS 0.102 (-0.009, 0.214) 0.071

IPM and NDS=3-5 vs. IPM or high NDS 0.042 (-0.005, 0.009) 0.083

IPM and NDS=3-5 vs. IPM and high VPT 0.067 (-0.05, 0.185) 0.260

IPM and NDS=3-5 vs.IPM or high VPT 0.013 (-0.053, 0.080) 0.696

IPM: indicator plaster method,VPT: vibration perception threshold, NDS: neuropathy disability score, high NDS: ≥6, high VPT: vibration perception threshold ≥25 Volts.

NDS and IPM: combined variable of participants with both tests abnormal;high NDS or IPM: combined variable of participants with abnormal either high NDS or IPM test; high VPT and IPM: combined variable of participants with both tests abnormal;high VPT or IPM: combined variable ofparticipants with abnormal either high VPT or IPM test; NDS=3-5 and IPM: combined variable of participants with both mild neuropathic signs and abnormal IPM.
